# First Report of *Meloidogyne ethiopica* and *M. javanica* in *Rumex* spp. in Rio Grande do Sul State, Brazil

**DOI:** 10.21307/jofnem-2019-060

**Published:** 2019-09-17

**Authors:** L. A. Yánez Márquez, M. Divers, W. R. Silva, J. V. de Araújo Filho, C. B. Gomes

**Affiliations:** 1 Departamento de Fitopatologia, Universidade Federal de Pelotas, 96010-900, Pelotas, RS, Brazil.; 2 Embrapa Clima Temperado, C.P. 403, Pelotas, RS 96001-970, Brazil.

**Keywords:** Brazil, identified, nematodes, root-knot

**Figure 1:**
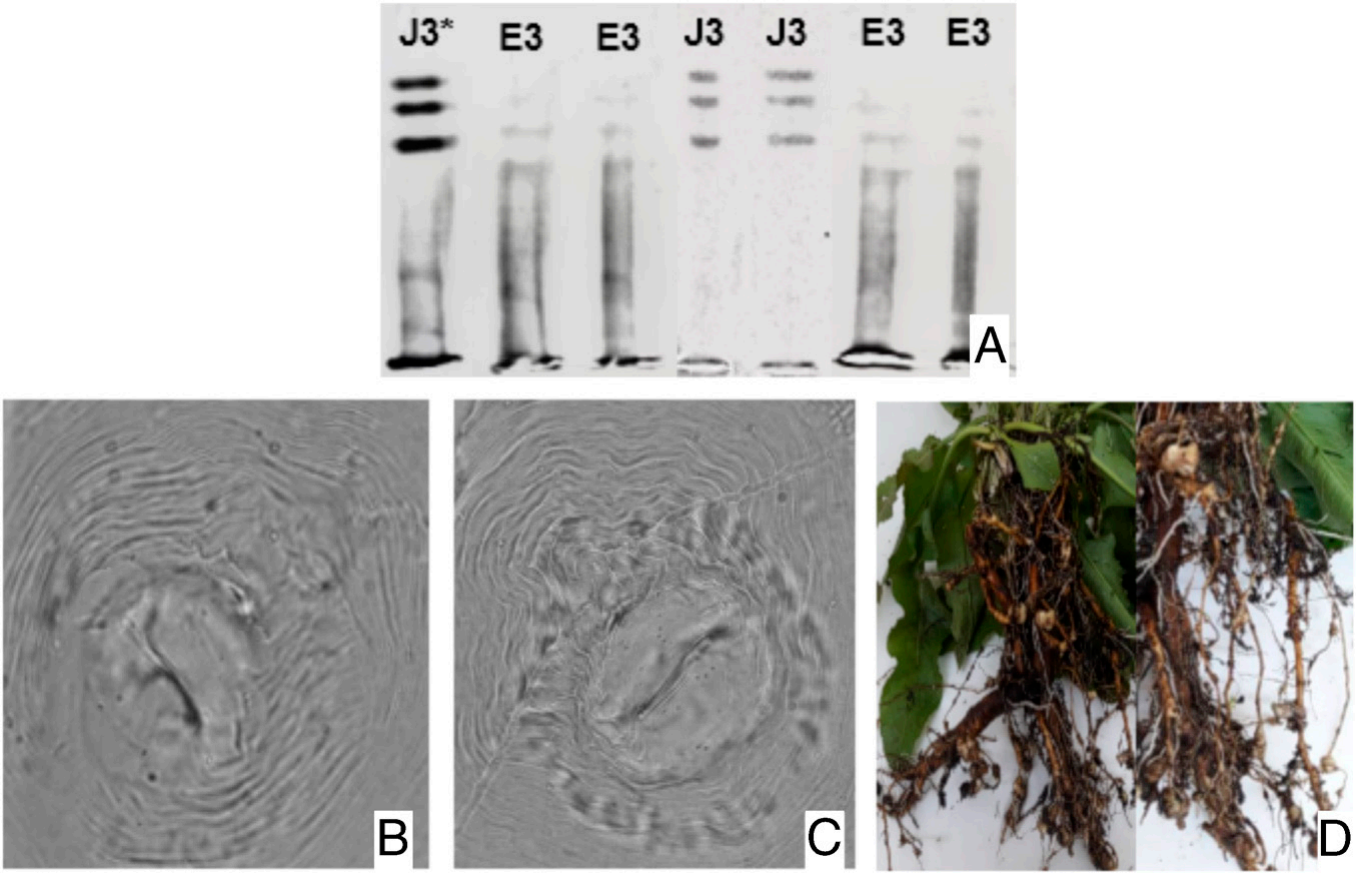
Appendix

*Rumex* spp. is a common weed in the southern of the Brazil, where it infests pastures, annuals crop and orchards. In September 2017, *Rumex* plants with root-knot disease were collected in the municipality of Pelotas, state of Rio Grande do Sul, Brazil. Initially, specimens were obtained by Coolen and D’Herde (1972) and later identified through morphological studies and esterase phenotypes (Carneiro and Almeida, 2001). Perineal patterns were consistent with *Meloidogyne ethiopica* (Whitehead, 1968) and *M. javanica* (Treub, 1885) (Chitwood, 1949). To *M. ethiopica*, perineal patterns were oval to squarish, with striae widely separated, smooth to wary and phasmids were distinct. Dorsal arch moderately high to high, rounded to squarish. In relation to *M. javanica*, perineal patterns were rounded with flattened dorsal arch, with distinct lateral lines, separating it into dorsal and ventral regions. From the esterase electrophoresis we obtained E3 (Rm:0.9;1.1;1.25) and J3 (Rm:1.0;1.25;1.4) phenotypes, typical from *M. ethiopica* (Randig et al., 2004) and *M. javanica*, respectively. The second-stage juveniles (*n* = 20) had the following morphometric characters: *L* = 392.3 (359 – 426.9) μm, stylet = 13 (12.1 – 13.5) μm, DGO = 2.4 (2 – 2.9) µm, tail length 58.6 (51.2 – 66.3) μm, hyaline tail terminus = 13.1 (12.2 – 14.3) μm, *a* = 22.2 (19 – 23.5) μm, and *c* = 6.7 (5.7 – 7.6) for *M. ethiopica*, and: *L* = 439.6 (438.2 – 511.4) μm, stylet = 14.6 (14.4 – 15.3) μm, DGO = 4 (2.6 – 4.9) μm, tail length = 55.6 (51.5 – 61.6) μm, hyaline tail terminus = 13.4 (11.2 – 18.5) μm, for *M. javanica*. Under greenhouse, *Rumex* plants were inoculated with 5,000 eggs plus J2s (*Pi*) of the original population of *M. ethiopica* and *M. javanica* (three replicates) and non-inoculated plants were included. After 90 days, plants showed root galls were evaluated and final population (*Pf*) was estimated. The reproduction factor (*RF* = *Pf*/*Pi*) was 50.40 and 43.40 for *M. ethiopica* and *M. javanica*, respectively. The non-inoculated plants did not present root galls. These results confirmed the nematode’s pathogenicity on *Rumex* spp. In 2003, it was the first record of *Meloidogyne ethiopica* in Kiwi (*Actinidia deliciosa*) fruit plants in Serra Gaúcha region (Carneiro et al., 2003). In Pakistan, Ahmad et al. (2015) reported occurrence of *M. javanica* on *Rumex crispus*, but we did not found record of *M. ethiopica*. Gharabadiyan et al. (2012) considered *Rumex acetosa* a good host only for *M. arenaria* race 2. *Rumex acetosella* has been classified as susceptible to *M. javanica* (Ansari et al., 2019). To the best of our knowledge, this is the first report of *M. ethiopica* and *M. javanica* parasitizing *Rumex* spp. roots in Brazil. This finding has a great importance, since to predict one host potential of nematodes in agricultural areas (Fig. 1).
